# Characterization of bursa subacromialis-derived mesenchymal stem cells

**DOI:** 10.1186/s13287-015-0104-3

**Published:** 2015-06-03

**Authors:** Andre F. Steinert, Manuela Kunz, Patrick Prager, Sascha Göbel, Ludger Klein-Hitpass, Regina Ebert, Ulrich Nöth, Franz Jakob, Frank Gohlke

**Affiliations:** Julius-Maximilians-University Würzburg, Department of Orthopaedic Surgery, König-Ludwig-Haus, Orthopaedic Center for Musculoskeletal Research, Julius-Maximilians-University Würzburg, Brettreichstr. 11, D - 97074 Würzburg, Germany; University of Duisburg-Essen, Center for Medical Biotechnology, BioChip Laboratory, Essen, Germany; Present address: Klinik für Schulterchirurgie, Rhön Klinikum AG, Bad Neustadt/Saale, Germany

## Abstract

**Introduction:**

The bursa subacromialis (BS) provides the gliding mechanism of the shoulder and regenerates itself after surgical removal. Therefore, we explored the presence of mesenchymal stem cells (MSCs) within the human adult BS tissue and characterized the BS cells compared to MSCs from bone marrow (BMSCs) on a molecular level.

**Methods:**

BS cells were isolated by collagenase digest from BS tissues derived from patients with degenerative rotator cuff tears, and BMSCs were recovered by adherent culture from bone-marrow of patients with osteoarthritis of the hip. BS cells and BMSCs were compared upon their potential to proliferate and differentiate along chondrogenic, osteogenic and adipogenic lineages under specific culture conditions. Expression profiles of markers associated with mesenchymal phenotypes were comparatively evaluated by flow cytometry, immunohistochemistry, and whole genome array analyses.

**Results:**

BS cells and BMSCs appeared mainly fibroblastic and revealed almost similar surface antigen expression profiles, which was CD44^+^, CD73^+^, CD90^+^, CD105^+^, CD106^+^, STRO-1^+^, CD14^−^, CD31^−^, CD34^−^, CD45^−^, CD144^−^. Array analyses revealed 1969 genes upregulated and 1184 genes downregulated in BS cells vs. BMSCs, indicating a high level of transcriptome similarity. After 3 weeks of differentiation culture, BS cells and BMSCs showed a similar strong chondrogenic, adipogenic and osteogenic potential, as shown by histological, immunohistochemical and RT-PCR analyses in contrast to the respective negative controls.

**Conclusions:**

Our in vitro characterizations show that BS cells fulfill all characteristics of mesenchymal stem cells, and therefore merit further attention for the development of improved therapies for various shoulder pathologies.

## Introduction

With an incidence of about 30 %, degenerative tears of the rotator cuff emerge as one of the most common musculoskeletal diseases in the older population [1, 2] with significant socio-economic impact [3–7]. Interestingly, it has been noted in the clinical area that localized reactions of the bursa subacromialis (BS) are evident in cases with rotator cuff tears [8], and that rotator cuff reconstructions reveal a lower success rate when surgical techniques are used that include radical resection of the BS [1]. Furthermore, in revision cases we have observed that the BS tissue is restored after complete surgical resection within approximately three to six months, indicating its high regenerative potential.

The BS represents extraarticular synovialis-like tissue that is anatomically located between the rotator cuff and the acromion and provides the gliding mechanism of the shoulder [9, 10]. Unfortunately, the BS has not received much attention by the scientific community yet. The subacromial bursa was traditionally regarded as the main source of subacromial pain, adhesions and inflammatory response in rotator cuff disease. This derives mainly from the concept of Duplay in the 19th century who influenced generations of orthopedic surgeons to remove the bursa during subacromial decompression and rotator cuff repair [11]. These ideas were supported by findings of increased levels of cytokines and nociceptors in subacromial impingement and rotator cuff tears [12–14]. Therefore, in the past most surgeons believed that the subacromial bursa acts mainly as a mediator of inflammation and tendon destruction rather than as a useful healing response for the repair of tendon lesions. Uhthoff and Sarkar first proved the healing potential of the subacromial bursa in human biopsies [15], and in an experimental animal model [16], which have been confirmed by others [17, 18]. However, the cellular mechanism of these findings has not been clarified yet, although BS cells have been recognized to express several morphogens and cytokines upon damage of the underlying rotator cuff tendon [19].

Mesenchymal stem cells (MSCs) have been isolated and extensively characterized from bone marrow [20, 21] and several mesenchymal tissues including bone [22], fat [23], cartilage [24], muscle [25], tendon [26, 27], ligament [28–30] and other sources [31, 32]. Given the self-regeneration capacities of the BS in vivo after surgical removal along with its localization adjacent to the rotator cuff, it was the purpose of this study to characterize the cells that reside within the BS, and secondly to explore their MSC properties compared to those of the well-characterized MSCs isolated from bone marrow (BMSCs).

## Materials and methods

### Tissue collection and cell isolation

Human BS tissues were harvested aseptically from 10 male 42- to 58-year old patients with degenerative tears of the rotator cuff undergoing reconstruction surgery (after informed consent and as approved by the local institutional review board of the University of Würzburg). The BS tissues were then rinsed twice with serum-free Dulbecco’s modified Eagle’s medium (DMEM)/F-12 media (PAA Laboratories, Linz, Austria) containing 1 % penicillin/streptomycin (PAA Laboratories). A small part of the tissues was reserved for histology, while the rest was minced to 1-2 mm^3^ pieces and placed in 0.1 % collagenase 1/3 solution (Life Technologies GmbH, Darmstadt, Germany). The recovered cells from the digest solution were plated in monolayer cultures in DMEM/F-12 media containing 10 % fetal bovine serum (Life technologies GmbH) and 1 % penicillin/streptomycin.

BMSCs were isolated from surgical waste of 10 male 45- to 65-year old patients undergoing total hip arthroplasty surgery after informed consent, and as approved by the Institutional Review Board of the University of Würzburg as described previously [22]. Briefly, bone-marrow reamings were harvested aseptically, resuspendend in DMEM/F-12 (PAA Laboratories), filtered through a 40 μm filter (BD Biosciences, Heidelberg, Germany), and plated in tissue culture flask. Non-adherent cells were removed after two days, and attached cells were washed with PBS, cultured in complete medium for 10 to 14 days to a subconfluent state, with medium changes every 3 to 4 days. Second to third passage cells were used for all experiments.

For analysis of chondrogenic differentiation, the BS and BMSC populations were expanded in the presence of 10 ng/ml fibroblast growth factor (FGF)-2 (PeproTech, Hamburg, Germany) as recommended [33].

### Cell proliferation assay

Proliferation rates of BS cells and BMSCs were determined by luminometrical measurements of adenosine-5′-triphosphate (ATP) activity using the CellTiter-Glo® Luminescent Cell Viability Assay (Promega GmbH, Mannheim, Germany) according to the manufacturer’s instructions. In short, 1,000 first passage cells per well were seeded in 96-well plates and cultured in 100 μl of complete medium for 17 days with media changes every second day. On days 1, 3, 5, 7, 9, 12, and 17 the luminescence of ten wells per donor and cell type was determined by addition of an equal volume of Cell Titer-Glo® reagent to the cells, incubation at room temperature for five minutes, and final detection of the luminescence signal for 0.1 seconds using an Orion II Luminometer (Berthold Detection Systems, Pforzheim, Germany). A total of five donors for each cell type were included.

### Flow cytometrical analyses

Flow cytometrical analyses were performed as previously described [28]. Briefly, monolayer BS cells and BMSCs from three different donors were detached from the culture flasks using ethylenediaminetetraacetic acid, suspended in PBS with sodium azide and transferred to 96-well V-bottom plates. After centrifugation at 400 g for three minutes, the supernatant was discarded. The cell pellets were then carefully washed twice with blocking buffer. For staining of intracellular antigens [alkaline phosphatase (ALP), FGF] an additional incubation step with Flow Cytometry Permeabilization Buffer I (R&D Systems, Heidelberg, Germany) was added. Following incubation at 4 °C for 30 minutes direct single- or multicolor immunofluorescent staining was performed with either 100 μl of an antigen-specific fluorescent monoclonal antibody or an immunoglobulin isotype control. After incubation at 4 °C for 30 minutes, the samples were centrifuged, and washed four times in 4 °C cold PBS with sodium azide. The prepared samples were either stored in 2 % paraformaldehyde or analyzed directly after preparation using a Cryonics FC 500 flow cytometer (Beckman Coulter). Monoclonal antibodies for CD34, CD53, CD73, CD90, CD105, CD106, CD133, CD144, CD166, ALP, FGF and Stro-1 conjugated with either allophycocyanin (APC), fluorescein isothiocyanate (FITC) or phycoerythrin (PE) were purchased from AbD Serotec, Beckman Coulter, BD Biosciences, Dako or R&D Systems. Non-specific monoclonal antibodies for each fluorochrome used, served as negative controls. Marker specifications are listed in Table 1.

**Table 1:** Expression of cell surface antigens and secreted proteins in BS cells and BMSCs

Antigen	Manufacturer	Label	Marker specification	Positive cells (%)	
				BS cells	BMSC
CD34	Beckman Coulter	PE	Hematopoietic stem cell marker, cell adhesion	7.69 (+)	1.05 (–)
CD53	AbD Serotec	FITC	Osteoblast and osteoclast signal transduction	0.00 (–)	0.14 (–)
CD73	BD Biosciences	PE	Mesenchymal, epithelial and endothelial cell marker	97.46 (+++)	98.67 (+++)
CD90	Beckman Coulter	PE	Fibroblast, stromal and hematopoietic stem cell marker	95.18 (+++)	98.37 (+++)
CD105	Beckman Coulter	PE	Mesenchymal and erythroid progenitor cells	98.05 (+++)	99.10 (+++)
CD106	AbD Serotec	FITC	Cell adhesion	4.09 (–)	55.55 (++)
CD133	Beckman Coulter	PE	Hematopoietic stem cell marker	0.14 (–)	1.20 (–)
CD144	AbD Serotec	FITC	Endothelial cells, cell adhesion	0.01 (–)	0.58 (–)
CD166	BD Biosciences	PE	Mesenchymal, epithelial stem cells, fibroblasts, monocytes, cell adhesion	88.06 (++)	98.42 (+++)
ALP	R&D Systems	APC	Alkaline phosphatase	22.79 (+)	55.50 (++)
FGF	R&D Systems	FITC	Fibroblast growth factor	45.06 (+)	42.64 (+)
Stro1	Dako	FITC	Mesenchymal stem cell marker	0.15 (–)	0.68 (–)

*+++* marker expression on >95 % of the cells, *++* marker expression on 50-95 % of the cells, *+* marker expression on 5-50 % of the cells, *-* marker expression on <5 % of the cells

*ALP* alkaline phosphatase, *APC* allophycocyanin, *BMSC* bone marrow-derived MSC, *BS* bursa subacromialis, *CD* cluster of differentiation, *FGF* fibroblast growth factor, *FITC* fluorescein isothiocyanate, *MSC* mesenchymal stem cells, *PE* phycoerythrin

### RNA isolation

RNA from three different BS and five different BMSC donors was extracted using the RNeasy extraction kit (Qiagen GmbH, Hilden, Germany) according to the manufacturer’s instructions. Synthesis of cDNA was performed with a total of 1 μg purified RNA using random hexamers (Life Technologies GmbH) and reverse transcriptase (RT) (Bioline GmbH, Luckenwalde, Germany) as indicated by the suppliers. RT-PCR was performed in a reaction volume of 50 μl containing 100 ng of synthesized cDNA, Taq DNA polymerase (Bioline GmbH) as well as target specific sense and antisense primers listed in Table 2, which also provides a summary of the primer specific annealing temperatures, optimal cycle numbers and expected fragment sizes. The resulting PCR products were separated by agarose gel electrophoresis containing 1.5 % agarose and 0.1 μg/ml ethidium bromide. Since the elongation factor 1α (EF1α) is a well-known housekeeping gene, it served as an internal control for normalization of gene expression.

**Table 2:** Primer sequences and PCR conditions

Gene	Oligonucleotide primer sequence	Number of cycles	Annealing temp. (° C)	Product size (bp)
Verfication of array data				
PRG4	S: 5′—GCTTGCACCCACCACCACCA—3′	38	60	210
	A: 5′—AGCTCCTTGGGGGCAGGCTT—3′			
FGF18	S: 5′—GTGGGGAAGCCCGATGGCAC—3′	35	62	208
	A: 5′—GAAGCTCCGGCTGCCCCTTG—3′			
FGF9	S: 5′—AATGTGCCCGTGTTGCCGGT—3′	35	60	421
	A: 5′—GCCTTCCAGTGTCCACGTGCT—3′			
Meox	S: 5′—CCAACTGGCACCTCCCGCAG—3′	37	62	204
	A: 5′—CCGCAGGTGACAGTGCCTGG—3′			
WISP3	S: 5′—CTGTGTTACATTCAGCCTTGCGAC—3′	29	54	337
	A: 5′—CTTGGTTTTACAGAATCTTGAGCTC—3′			
CD200	S: 5′—TGGCAGCAGTGGTGCTGTGC—3′	40	60	354
	A: 5′— AGACGGTGAGGCAGGCCGTT—3′			
BSP	S: 5′—AATGAAAACGAAGAAAGCGAAG—3′	33	54	450
	A: 5′—ATCATAGCCATCGTAGCCTTGT—3′			
FOXP2	S: 5′—AATCGCTGCCTCAAGCTGGC—3′	30	61	493
	A: 5′—GGTTTGGGCTCTGAGGGTCGC—3′			
MUC1	S: 5′— AATGAATGGCTCAAAACTTGG —3′	30	60	231
	A: 5′— CACTAGGTTCTCACTCGCTCAG —3′			
Differentiation assays				
Chondrogenic marker genes				
AGN	S: 5′—TGAGGAGGGCTGGAACAAGTACC—3′	30	54	392
	A: 5′—GGAGGTGGTAATTGCAGGGAACA—3′			
DEC	S: 5′—AATTGAAAATGGGGCTTTCC—3′	27	53	220
	A: 5′—GCCATTGTCAACAGCAGAGA—3′			
FM	S: 5′—CTTACCCCTATGGGGTGGAT—3′	35	54	389
	A: 5′—GTACATGGCCGTGAGGAAGT—3′			
SOX9	S: 5′—ATCTGAAGAAGGAGAGCGAG—3′	35	58	263
	A: 5′—TCAGAAGTCTCCAGAGCTTG—3′			
IHH	S: 5′—GAGGAGTCCCTGCATTATGA—3′	30	54	321
	A: 5′—CAGGAAAATGAGCACATCGC—3′			
COL II	S: 5′—TTTCCCAGGTCAAGATGGTC—3′	35	58	374
	A: 5′—CTTCAGCACCTGTCCACCA—3′			
Osteogenic marker genes				
ALP	S: 5′—TGGAGCTTCAGAAGCTCAACACCA—3′	25	51	454
	A: 5′—ATCTCGTTGTCTGAGTACCAGTCC—3′			
COL I	S: 5′—GGACACAATGGATTGCAAGG—3′	30	54	461
	A: 5′—TAACCACTGCTCCACTCTGG—3′			
Cbfa1	S: 5′—ACAGATGATGACACTGCCACC—3′	30	55	324
	A: 5′—CATAGTAGAGATATGGAGTGCTGC—3′			
Adipogenic marker genes				
LPL	S: 5′—GAGATTTCTCTGTATGGCACC—3′	30	51	276
	A: 5′—CTGCAAATGAGACACTTTCTC—3′			
PPARγ2	S: 5′—GCTGTTATGGGTGAAACTCTG—3′	30	51	351
	A: 5′—ATAAGGTGGAGATGCAGGCTC—3′			
Internal control				
EF1α	S: 5′—AGGTGATTATCCTGAACCATCC-—3′	24	54	234
	A: 5′—AAAGGTGGATAGTCTGAGAAGC—3′			

*A* antisense, *AGN* aggrecan, *ALP* alkaline phosphatase, *bp* base pair, *BSP* integrin-binding sialoprotein, *Cbfa1* core binding factor alpha 1, *CD200* cluster of differentiation 200, *COL I* collagen type I, *COL II* collagen type II, *DEC* decorin, *EF1α* elongation factor 1α, *FGF* fibroblast growth factor, *FM* fibromodulin, *FOXP2* forkhead box P2, *IHH* indian hedgehog, *LPL* lipoprotein lipase, *Meox* mesenchyme homeobox 2, *MUC1* mucin 1, *PPARγ2* peroxisome proliferator-activated receptor gamma 2, *PRG4* proteoglycan 4, *S* sense, *SOX9* SRY (sex determining region Y)-box 9, *temp.* temperature, *WISP3* WNT1 inducible signalling pathway protein 3

### Genome-wide gene expression profiling of bursa cells and BMSCs

For genome-wide gene expression profiling, hybridization experiments were performed and analyzed using Affymetrix Gene Chips HG-U133 Plus 2.0 (54,000 probesets for 47,400 transcripts and 38,500 genes, High Wycombe, UK) as described previously [34]. Total RNA expression of three different BS and five individual BMSC preparations were analyzed using the Affymetrix Gene Chip Scanner 3000, the Affymetrix GeneChip Operating Software 1.4, and comparatively evaluated using the significance analysis of microarrays (SAM) approach [35].

To assess differentially expressed genes between BS and BMSC groups, pre-defined conditions were established: the number of “present” calls for a given gene had to be greater than 50 % in at least one of the groups and only those genes were taken into account that displayed a fold change (FC) less than 0.5 and more than 2. Probesets which showed an FC between 0.5 and 2 were stated as “not differentially expressed”. In order to obtain reliable data, the q-value, i.e. false discovery rate had to be less than 10 %. For heatmap generation and to identify significantly overrepresented gene clusters, Gene Ontology (GO) analysis and mapping was performed with all differentially expressed probesets by using the web service for microarray data analysis CARMAweb [36]. Confirming RT-PCR analyses from five up- and five downregulated genes were performed using primers and PCR conditions as listed in Table 2.

### Chondrogenesis

Chondrogenic differentiation was performed in high density cultures as previously described [37]. Briefly, 3 × 10^5^ cells were resuspended in 0.5 ml chondrogenic medium DMEM high glucose (PAA Laboratories) supplemented with 50 μg/ml L-ascorbic acid 2-phosphate, 100 nM dexamethasone, 100 μg/mL pyruvate, 40 μg/ml L-proline, 1 % ITS+ (all Sigma Aldrich GmbH, Munich, Germany), 10 ng/ml transforming growth factor-β1 (TGF-β1) (R&D Systems) and pelleted. The generated aggregates were maintained in chondrogenic medium for three weeks with medium changes every two to three days. Control pellets were maintained without TGFβ1 supplementation. For histological and immunohistochemical analyses, aggregates were fixed in 4 % paraformaldehyde, dehydrated in a graded series of alcohols and embedded into paraffin. Thick sections (4 μm) were mounted on slides, washed with xylene, and rehydrated in a series of graded alcohols. Stainings were performed using standard protocols for matrix-associated proteoglycans using alcian blue. Immunohistochemical detection of collagen type II (COL II) was performed as described in detail previously [37] using a primary monoclonal COL II antibody (Acris Antibodies GmbH, Herford, Germany), while negative controls were treated with mouse serum instead. Detection of the staining was done using the Biogenex Super Sensitive^TM^ Link-Label IHC Detection System (DCS Innovative Diagnostic Systems, Hamburg, Germany) according to the manufacturer’s instructions.

Expression of chondrogenic marker genes was performed using RT-PCR analyses with the lineage specific primers and PCR conditions summarized in Table 2. Six pellets per group were initially frozen in liquid nitrogen, ground using pellet pestles and added to 1 ml of Trizol reagent (Life Technologies GmbH), with an additional purification step using RNeasy separation columns (RNeasy kit; Qiagen, Hilden, Germany) according to the manufacturer’s instructions. Three different donors for each cell type were analyzed.

### Osteogenesis

For osteogenic differentiation 1 × 10^5^ cells per cm^2^ were seeded in four-well chamber slides and 25 cm^2^ culture flasks (both Nunc). Once the cell layers reached confluency, osteogenic differentiation was induced by cultivation in complete osteogenic DMEM high glucose medium (PAA) supplemented with 100 nM dexamethasone, 50 μg/ml L-ascorbic acid 2-phosphatase, 10 mM β-glycerophosphate (all Sigma Aldrich GmbH) as well as 25 ng/ml recombinant human BMP2 (R&D Systems GmbH). Cultures maintained in control medium (lacking the above listed supplements) served as a negative control for differentiation. Osteogenesis was conducted for three weeks with medium changes every two to three days. Histological analyses were performed by staining for alkaline phosphatase (ALP) using the Leukocyte ALP Staining Kit (Sigma Aldrich GmbH) according to the manufacturer's instructions. Matrix mineralization was evaluated by staining with alizarin red, as described previously [22]. The parameters for expression analysis of osteogenic marker genes are listed in Table 2 and semi-quantitative RT-PCR analyses were performed as described above. Three different donors for each cell type were analyzed.

### Adipogenesis

Adipogenic differentiation required the initial seeding of cells at a density of 1 × 10^5^ cells per cm^2^. After the cell layer reached about 50-70 % confluency, the cells were further cultivated in adipogenic induction medium, consisting of complete DMEM high glucose supplemented with 1 μM dexamethasone, 1 μg/ml insulin, 0.5 mM 3-isobutyl-1-methylxanthine (IBMX) and 100 μM indomethacin for three weeks with media changes every two to three days. Control cells were cultivated in supplement-free adipogenic control medium. Histological analyses using oil red O staining were conducted to detect formation of lipid droplets. RT-PCR analyses were performed as stated previously using lineage specific primers and PCR conditions as summarized in Table 2. Three different donors for each cell type were analyzed.

### Histology and immunohistochemistry of BS cells and tissue

Following fixation with 4 % paraformaldehyde, paraffin embedding, sectioning to 4 μm and rehydration of BS tissues was performed as described previously [28]. A general histological assessment of the sections was given using hematoxylin and eosin (H&E) staining, whereas mucines were stained using periodic acid-Schiff (PAS) staining and collagenous structures were detected by Azan, Masson-Goldner trichrome (MG), or van Gieson (VG) staining as described in detail earlier [28]. Immunohistochemical detection of surface antigens was performed as described above making use of antibody dilutions specific for CD44 (1:200), CD90 (1:25), CD105 (1:50) and the Stro1 (1:20) antigen (all Dako Deutschland GmbH, Hamburg, Germany) after tissue processing. For more detailed analyses of the intracellular distribution of positive Stro1 staining we also employed a fluorescein isothiocyanate (FITC) labeled Stro1 antibody with counterstaining of the nuclei using 4, 6-diamidino-2-phenylindole (DAPI) and phase contrast (bright field) and fluorescence microscopy (1:10; Santa Cruz Biotechnology Inc., Heidelberg, Germany).

### Statistical analyses

The numerical data are expressed as means +/- standard error (SEM). Determination of the statistical significance between groups was performed using student’s *t*-test, or the Mann-Whitney *U* test as indicated.

## Results

### Cell morphology, proliferation and surface antigen expressions of BS cells and BMSCs

**Fig. 1:**
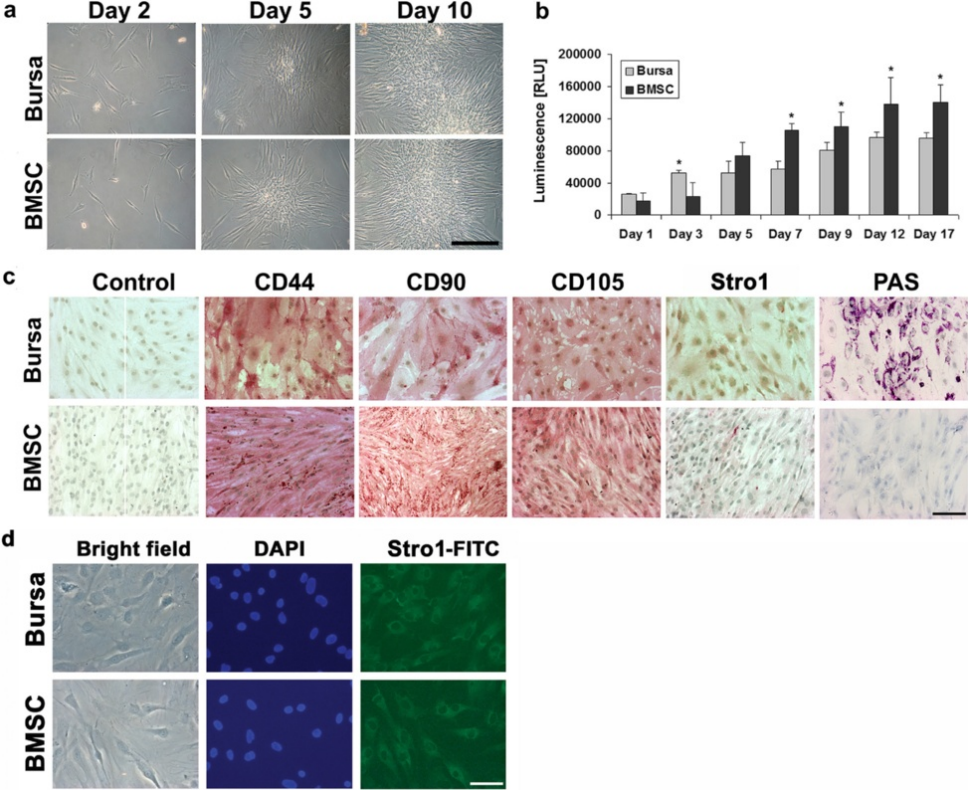
Morphology, proliferation and surface antigen analysis of BS cells and BMSCs. **a** BS cells have a fibroblast-like morphology typical for mesenchymal progenitor cells like BMSCs. Scale bar = 200 μm. **b** Comparative cell proliferation rates as determined by measurement of ATP activity showed an increase of ATP activity in BS cells at early and a decrease at late time points as compared with BMSCs. Proliferation of both cell types increased over time. A total of five donors were included with ten measurements for each time point and cell type. Significant differences between the two groups are indicated by asterisks as determined by *t*-testing. **c** Immunohistochemical analysis was verified by the use of mouse serum instead of primary antibodies. The mesenchymal cell surface antigens CD44, CD90 and CD105 could be detected on BS cells as well as on BMSCs and exhibit similar staining intensities on both cell types whereas Stro1 intensities were low for BS cells and BMSCs. Periodic acid-Schiff (PAS) staining for mucines was exclusively positive in BS cells whereas BMSCs where negative. Scale bar = 100 μm. **d** For more detailed analyzes of the Stro1^+^ areas in BS cells and BMSCs, immunostaining with a FITC-labeled Stro1 antibody and DAPI counterstain of the nuclei was performed, revealing positive staining at similar levels for both cell types at high resolution. Scale bar = 25 μm. *BMSCs* bone-marrow derived mesenchymal stem cells *BS* bursa subacromialiss *DAPI* 4, 6-diamidino-2-phenylindole *FITC* fluorescein isothiocyanate

Cells isolated from human BS as well as BMSCs have a similar spindle-shaped, fibroblast-like morphology (Fig. 1a), and formed colonies upon adherent culture (Fig. 1a; day 5). Proliferative analysis using the Cell Titer Glo® Luminescent Cell Viability Assay showed an increase of ATP activity over time with equal activity dimensions for both cell types. Nevertheless time-dependent differences were observed as BS cells have significantly higher proliferation rates on day 3 while BMSCs surpass BS cell proliferation at day 7-17 (Fig. 1b).

Immunohistochemical detection of the MSC-associated surface markers CD44, CD90 and CD105 was strongly and equally positive for BS cells and BMSCs (Fig. 1c). Staining for the Stro1 antigen in contrast resulted in weak but positive signals for both cell types (Fig. 1c). Control cells incubated with mouse serum instead of the respective primary antibodies showed no staining and therefore validated the immunohistochemical investigations. Additionally PAS staining for the histological detection of mucines showed positive signals for BS cells, while BMSC monolayer cells remained unstained (Fig. 1c). For a more detailed analysis of the distribution of STRO1^+^ areas in BS cells and BMSCs, immunostaining with a FITC-labeled STRO1 antibody and DAPI counterstain of the nuclei was performed, revealing positive staining at similar levels for both cell types in the respective phase contrast (bright field) and corresponding fluorescence microscopy images (Fig. 1d).

Further in-depth analyses of cell surface markers using flow cytometry revealed that expression of the antigens CD73, CD90 and CD105 was strongly positive on BS cells and BMSCs (Table 1). Furthermore, CD166 is highly expressed on BMSCs while BS cells have a small subset of cells, which are negative for the respective antigen. In contrast, CD53, CD133, CD144 as well as the Stro1 antigen were not, or almost not, detectable on both cell types. Heterogeneous cell distributions were found for FGF, which is expressed by half of the BS cells and BMSCs. Further differences were found concerning the secretion of ALP: while about half of the BMSCs were positive, only a quarter of the BS cells showed positive signals. The expression of CD34 was negative in BMSCs, while a small subset of BS cells (7.69 %) expressed the respective antigen. On the other hand, BS cells showed no expression of CD106, which is in contrast to the moderate expression (55.55 %) of this marker in BMSCs.

### Microchip hybridization of RNA from BS cells and BMSCs

**Fig. 2:**
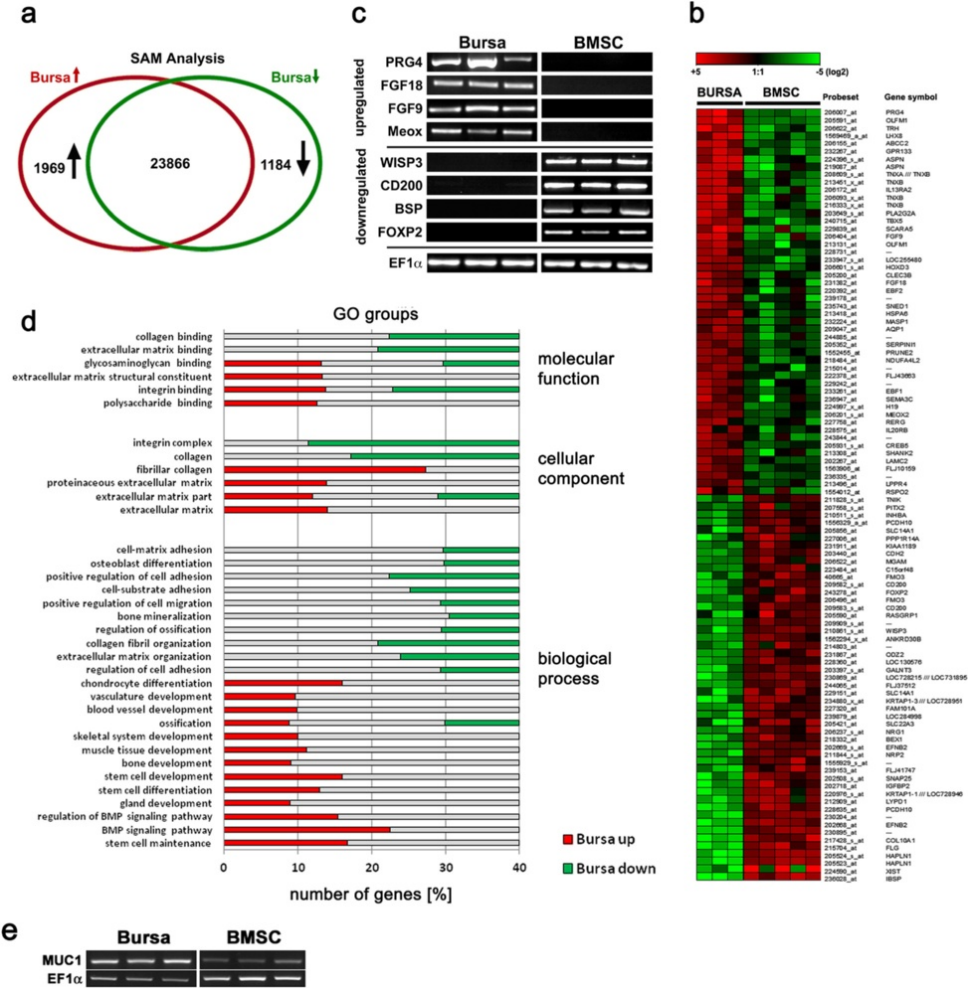
Comparison of microchip hybridizations for RNA from BS cells and BMSCs. **a** Significance analysis of microarray (SAM) revealed the number of probesets, which were upregulated (red circle) and downregulated (green circle) in BS cells as compared to BMSCs as well as the number of unregulated probesets (intersection). **b** Regulation of selected probesets from three BMSC donors and three BS cell donors. Upregulated probesets are represented by red areas, downregulated by green ones with light colors indicating stronger regulation than darker colors. **c** Validation of the microarray results using RT-PCR with three biological replicates for each cell source for the genes fibroblast growth factor (FGF) 9 and 18, proteoglycan 4 (PRG4), mesenchyme homeobox 2 (Meox), CD200, forkhead box P2 (FOXP2), integrin-binding sialoprotein (BSP), WNT1 inducible signaling pathway protein 3 (WISP3) and EF1α serving as normalization control. **d** Gene Ontology (GO) analysis of all differentially expressed probesets (3,153 altogether) identified significantly enriched “molecular function”, “cellular component” and “biological process” GO clusters. Shown are various sub-clusters identified in each major GO cluster. **e** RT-PCR analyses of the expression of the epithelial marker mucin 1 (MUC1) in three different donors for each cell type, with EF1α serving as normalization control. *BMSCs* bone-marrow derived mesenchymal stem cells *BS* bursa subacromialiss

Comparative microchip hybridization analysis of the RNA from three donors of BS cells, or five donors of BMSCs revealed a different gene regulation pattern for the two cell types. Significance analysis of microarray (SAM) is summarized in Fig. 2a, showing a Venn diagram, depicting the upregulation of 1,969 probesets in BS cells as compared to BMSCs, and the downregulation of 1,184 probesets in contrast, as well as a total of 23,866 unregulated probesets. The 50 most up- and down-regulated probesets are depicted in a heatmap (Fig. 2b), showing remarkable expression differences between BMSCs and BS cells, for example in the chondrocyte-associated genes proteoglycan 4 (PRG4), asporin (ASPN), hyaluronane and proteoglycan link protein 1 (HAPLN1), as well as in genes associated with junctions and adhesion, such as laminin γ2 (LAMC2), R-spondin 2 (RSPO2) and protocadherin 10 (PCDH10). Validation of the microchip hybridization was conducted using RT-PCR (Fig. 2c). The genes for fibroblast growth factor (FGF) 9 and 18, proteoglycan 4 (PRG4) and mesenchyme homeobox 2 (MEOX) were upregulated in BS cells compared to BMSCs (Fig. 2c). In contrast the genes for the markers cluster of differentiation 200 (CD200), forkhead box P2 (FOXP2), integrin-binding sialoprotein (BSP) and WNT1 inducible signaling pathway protein 3 (WISP3) were highly expressed in BMSCs but nearly not detectable in BS cells and confirmed the results of the microarray analyses (Fig. 2c). In order to identify a molecular pattern behind this transcriptomal shift, we performed Gene Ontology (GO) analysis with all 3,153 differentially expressed probesets. As a result, these differentially expressed probesets were assigned to “molecular function”, “cellular component” and “biological process” and GO clusters composed of various sub-clusters such as “stem cell development”, “stem cell differentiation”, “fibrillar collagen” and classes including “extracellular matrix” or “fibrillar collagen”, among others, as indicated in Fig. 2d. To further explore functional differences between BS cells and BMSCs, expression of the epithelial marker for mucus secretion mucus 1 (MUC1) was analyzed, and shown to be expressed in both cell types with somewhat stronger bands in the BS cells, indicating a role of this marker not only in epithelial cells but also in stem cells of mesenchymal origin.

### Chondrogenic differentiation of BS cells and BMSCs

**Fig. 3:**
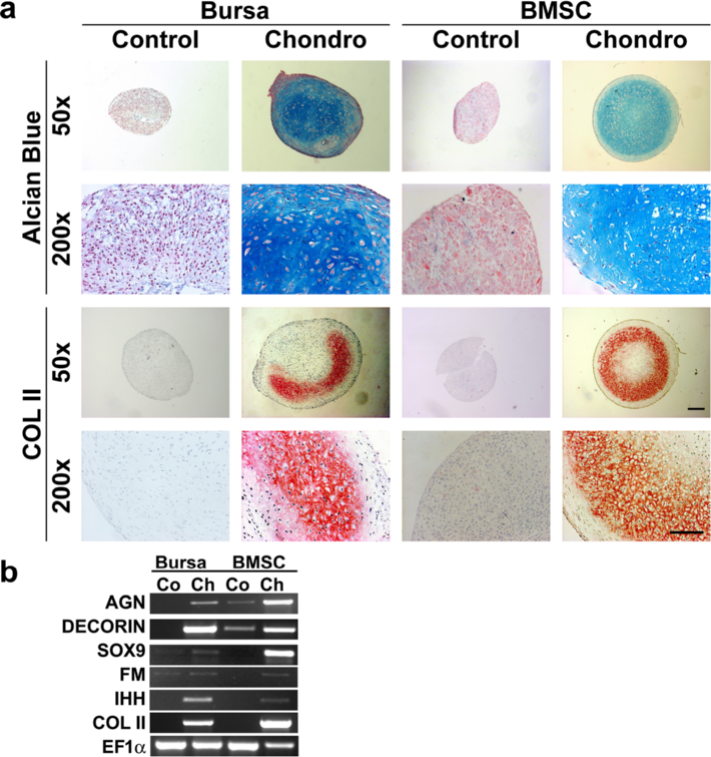
Chondrogenic differentiation of BS cells and BMSCs. Cells cultivated in chondrogenic medium showed strong staining for proteoglycans determined by positive alcian blue staining (Alc Blue) and were also positive for collagen type II (COL II) in BS cells as well as in BMSC compared to cells cultivated in control medium (**a**). Left scale bar = 200 μm; right scale bar = 100 μm. **b** Expression of chondrogenic marker genes was evaluated using RT-PCR. Cultivation in the presence of chondrogenic medium (Ch) resulted in an increased expression of aggrecan (AGN), decorin (DEC), SRY (sex determining region Y)-box 9 (SOX9), indian hedgehog (IHH), and collagen type II (COL II) as compared to untreated cells (Co) for both cell types. Expression levels of the housekeeping gene elongation factor 1α (EF1α) are shown in the last row. Representative images from three different donors are shown. *BMSCs* bone-marrow derived mesenchymal stem cells *BS* bursa subacromialiss

Differentiation along the chondrogenic lineage resulted in the deposition of proteoglycans in the extracellular matrix as determined by alcian blue staining for BS cells and BMSCs (Fig. 3a) with staining being less intense at the outer rim of the aggregates. Alcian blue staining of pellets maintained in chondrogenic control medium on the other hand showed a vanishingly low proteoglycan secretion as compared to the induced aggregates. These data are consistent with the mRNA expression levels of the proteoglycans aggrecan (AGC), decorin (DEC), and fibromodulin (FM) which are upregulated in chondrogenic differentiated cells as compared to pellets cultured in chondrogenic control medium (Fig. 3b).

Immunohistochemical detection of COL II was positive for both cell types after chondrogenic induction, and negative for the control pellets (Fig. 3a). The staining was very prominent in the inner areas of the aggregates. Analysis of RNA expression validates the staining with COL II being exclusively expressed by chondrogenic differentiated pellets and not by the related controls (Fig. 3b). Furthermore, maintenance of BS cells and BMSCs in chondrogenic medium instead of control medium resulted in higher expression of the chondrogenic SRY transcription factor box9 (SOX9), and the prehypertrophy-related marker gene indian hedgehog (IHH), while both mRNAs were not expressed in the corresponding controls (Fig. 3b).

### Osteogenic differentiation of BS cells and BMSCs

**Fig. 4:**
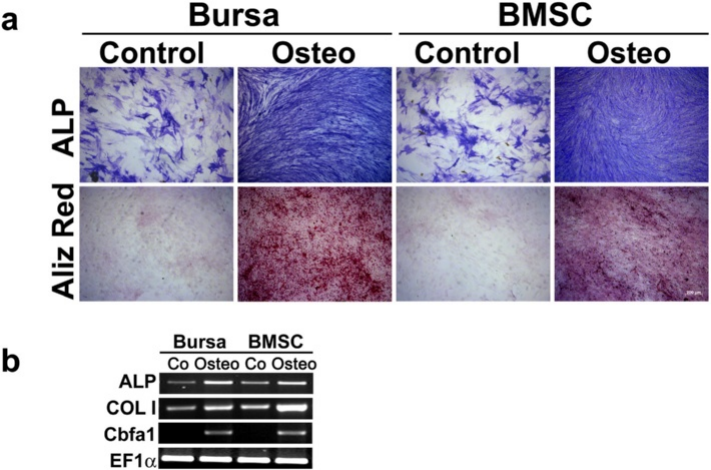
Osteogenic differentiation of BS cells and BMSCs. Cultivation of BS cells and BMSCs in osteogenic medium resulted in an increased activity of alkaline phosphatase (ALP) and beginning mineralization identified by alizarin red (Aliz Red) staining with similar staining intensities for BS cells and BMSCs (**a**). Scale bar = 200 μm. **b** The osteogenic marker genes ALP, collagen type I (COL I) and core binding factor α1 (Cbfa1) showed an increase in expression in cells cultured with osteogenic medium (Ost) as compared with those treated with control medium (Co). Elongation factor 1α (EF1α) served as a housekeeping gene for normalization of the expression values. Representative images from three different donors are shown. *BMSCs* bone-marrow derived mesenchymal stem cells *BS* bursa subacromialiss

Maintenance of BS cells and BMSCs in osteogenic medium resulted in increasing activities of the early osteogenic marker ALP in comparison to untreated control cells (Fig. 4a) with staining intensities being similar in both cell types. Alizarin red stainings show equal amounts of matrix mineralization in osteogenic differentiated BS cells and BMSCs, and nearly no staining in the respective control cells (Fig. 4a).

Expression of ALP mRNA is upregulated in osteogenic differentiated cells compared to cells maintained in osteogenic control medium for both cell types (Fig. 4b). Other osteogenic markers such as collagen type I (COL I) and Cbfa1 were also upregulated following differentiation along the osteogenic lineage, with Cbfa1 being exclusively expressed in differentiated cells of both cell types and not in the related controls (Fig. 4b).

### Adipogenic differentiation of BS cells and BMSCs

**Fig. 5:**
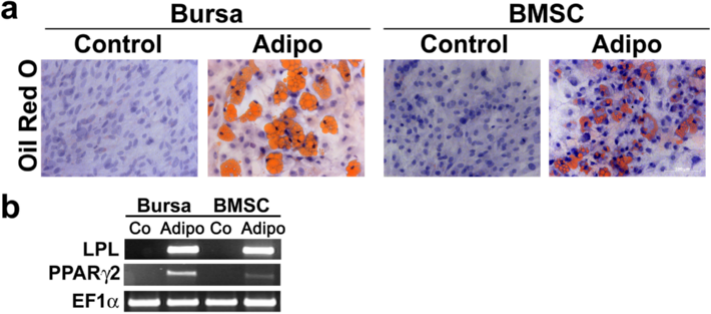
Adipogenic differentiation of BS cells and BMSCs. Cells cultivated in adipogenic medium showed formation of lipid droplets in BS cells and BMSCs as determined by Oil Red O staining. Cultivation in control medium in contrast did not result in droplet enrichment (**a**). Scale bar = 100 μm. **b** Expression of the adipogenic marker genes lipoprotein lipase (LPL) and peroxisome proliferator-activated receptor gamma 2 (PPARγ2) was increased in BS cells and BMSCs treated with adipogenic medium (Adi), but was not detectable in cells cultivated with control medium (Co). The housekeeping gene EF1α showed equal expression levels in all groups observed. Representative images from three different donors are shown *BMSCs* bone-marrow derived mesenchymal stem cells *BS* bursa subacromialiss.

Oil red O staining was used for histological detection of lipid droplets associated with adipogenic differentiated BS cells and BMSCs (Fig. 5a) showing formation of red lipid droplets in contrast to control cells, which lack staining. For BS cells and BMSCs no differences could be detected in RNA expression of mRNAs from lipoprotein lipase and PPARγ2, which were exclusively expressed in cells cultured with adipogenic medium and absent in the control cells (Fig. 5b).

### Histological and immunohistochemical characterization of tissue sections from bursa subacromialis

**Fig. 6:**
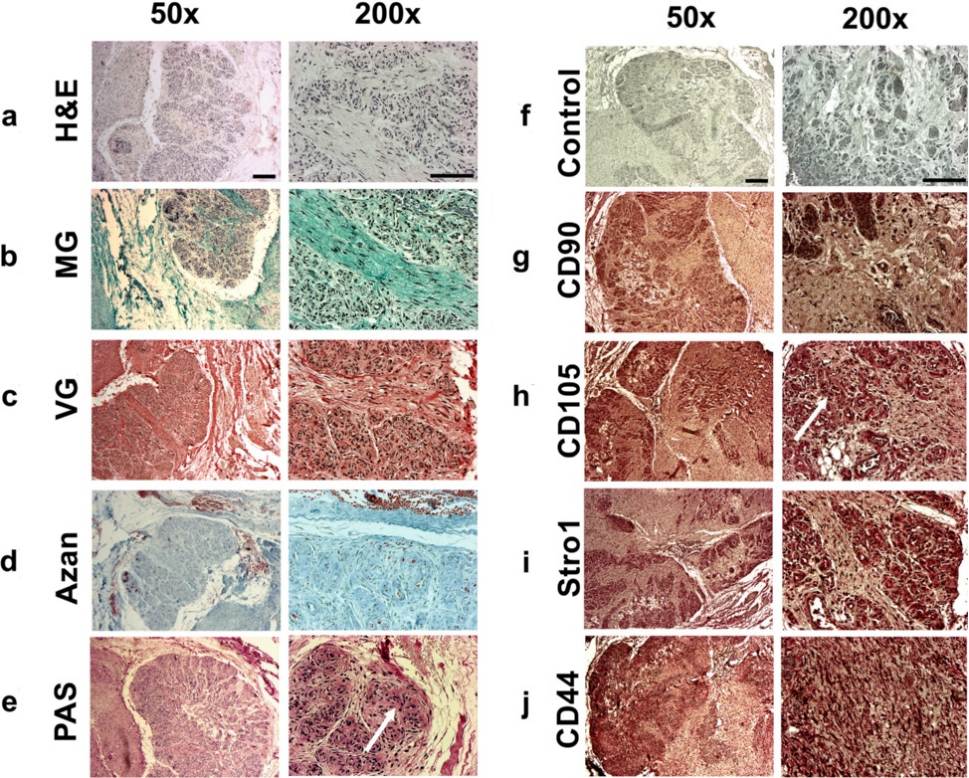
Histological and immunohistochemical characterization of paraffin embedded tissue sections from bursa subacromialis. Tissue sections were histologically analyzed and stained for general evaluation with **a** hematoxylin and eosin stain (H&E), stained positive for collagens using **b** Masson-Goldner trichrome (MG) staining, **c** Van Gieson (VG) staining or **d** Azan staining. **e** Detection of mucines was performed using Periodic-Acid-Schiff (PAS) staining. Immunohistochemical detection of surface antigens was negative for incubation with **f** mouse serum instead of the primary antibody serving as a negative control, but clearly positive for **g** CD44, **h** CD90, **i** CD105 and **j** Stro-1. Left scale bar = 200 μm; right scale bar = 100 μm. Representative images of six different donors are shown

For further characterization of native BS tissue, sections were stained using several histological methods and immunohistochemistry for detection of surface antigens characteristic for MSCs. H&E staining (Fig. 6a) illustrates the general cellular composition of the tissue with its structural units. Three different stains for the detection of collagenous fibers were used, showing positive staining for MG (Fig. 6b), VG (Fig. 6c) and Azan (Fig. 6d), thus verifying the rich presence of collagens within the bursa. Periodic acid-Schiff (PAS) staining was positive for the BS tissue revealing its gland-associated origin (Fig. 6e). Immunohistochemical analyses resulted in no staining for the mouse serum treated control cells (Fig. 6f), but stained positive for the mesenchymal markers CD44 (Fig. 6g), CD90 (Fig. 6h), CD105 (Fig. 6i), and also to some extent for the Stro1 antigen (Fig. 6j).

## Discussion

Bursae are saclike cavities situated in places in the body where friction would otherwise strongly occur, facilitating the gliding of tendons over solid surfaces [10]. Bursae in shoulder joints retain the ability to regrow after partial or total surgical removal according to our own clinical observations. The subacromial bursa (BS) vividly responds to rotator cuff injuries [8] and degeneration [19] and successfully augments rotator cuff repair surgery [15]. Therefore the purpose of the current study was to characterize the phenotype of BC cells compared to the well-characterized BMSCs. Our results provide evidence for the existence of a rich population of multipotent MSCs within the BS with similar fibroblastic appearance and almost similar proliferation profile compared with BMSCs (Fig. 1). This is consistent with findings of different MSC populations in the literature with differences in cell proliferation being attributed to different tissue sources and disease pathologies [38–40].

Characterization of cell surface markers of BS cells and BMSCs revealed the markers CD44, CD90, and CD105 positive by immunohistochemistry (Fig. 1c) and flow cytometry (Table 1) which is consistent with the abundant literature in the MSC field [28, 41, 42]. In contrast, the Stro1 antibody showed only a slight staining in immunohistochemistry and was not detectable at marked levels in either cell type using flow cytometrical methods. This might be an antibody-related effect [43] and corresponds with previous findings in ligament MSCs [28]. Marked differences between cell types could be seen in the expression of CD34, CD106 and ALP (Table 1), indicating that a subset of cells identified in BS could be CD34^+^ endothelial progenitors, while a larger amount of ALP^+^ bone progenitors is present in BMSCs [44–46]. Additionally, PAS staining of mucopolysaccharides could be detected in BS cells but not in BMSCs illustrating a major difference between the donor sources of the two mesenchymal cell types used, which did not impair the multilineage potential of the BS cells (Figs. 3, 4 and 5). This finding was supported by a strong expression of mucin marker MUC1 in BS cells (Fig. 2e). As this marker, however, was also present in BMSCs, we can speculated on the role of secretory pathway activation in stem cells of mesenchymal origin.

Not only did the immunohistochemical staining of monolayer cells show a strong correlation of BS cells and BMSCs, but detection of the same MSC markers, CD44, CD90, and CD105, showed that positive areas for these markers could be located on the fibrous as well as the mucinous portions of the BS tissue, indicating the presence of MSCs within different parts of the BS (Figs. 1c and 6). Notably, variability in staining intensity may reflect different stages of vascularity rather than different staining intensities in the stromal matrix of the bursa tissue. This corresponds to the finding of MSCs in the palatine tonsil, where stem cell niches could also be located in mucinous and fibrous regions of this gland [39]. Therefore, it remains to be seen in future experiments, whether the PAS^+^ subset of exocrine cells within the BS population exhibits a different multipotency profile compared to the PAS^-^ fraction of the BS population (Fig. 6), and also to explore the regenerative capacities of these cell types.

Differences between BS cells and BMSCs could be further resolved on a molecular level using analyses of the respective transcriptomes, indicating 7.29 % up- and 4.38 % downregulated probesets in BS cells compared to BMSCs (Fig. 2a). Interestingly, further examinations revealed that several cartilage and bone-associated genes (e.g. BSP, WISP3, COL X) were exclusively upregulated in BMSCs but not in BS cells, indicating the relevance of the tissue source in the evaluation of transcriptional profiles [34, 47]. This is in agreement with the finding of FGF9 and FGF18 expression in BS cells and not in BMSCs, with FGF9 being an inverse regulator of BMP2 [48], and FGF18 responsible for dedifferentiation of chondrocytes and fibroblast proliferation [49]. CD200, a regulator of macrophage activation and novel MSC marker [49, 50], was only confirmed for BMSCs but not for BS cells, holding the advantage to distinguish between both cell types. While microchip array analyzes are powerful screening tools, significant variation and room for interpretation of results has to be considered.

Apparently, the BS is often a site of pathology in impingement of the shoulder [1]. In this study, all BS cells were obtained from patients undergoing rotator cuff repair surgery as a result of degenerative full-thickness tears. It is known that the size of the tear has a direct influence on the inflammatory status of the adjacent bursa [13], as evidenced by the increasing levels of inflammatory cytokines, such as interleukin 1 (IL1), IL6, metalloproteases, tumor necrosis factor α [14, 51] and myofibroblast invasion [52]. Therefore, we cannot rule out that myofibroblasts from the underlying rotator cuff might have entered the bursa and added to the BS cell population examined, as BS tissues from healthy donors could not be retrieved, which is a limitation of this study. Indeed, it is not possible to know where these inflammatory bursal cells may have migrated from, or whether they existed in the bursa to start. Thus, future studies are necessary to understand better the impact of the inflammatory microenvironment on MSCs for their application in therapeutic protocols.

Only recently, the BS has been identified by others as a novel source of MSCs within the shoulder, confirming their differentiation potential into the chondrogenic, osteogenic and adipogenic lineage and expression of the surface antigens CD73 and 90 [53]. Furthermore BS cells were also found to express the surface markers CD29 and platelet-derived growth factor receptor-beta (PDGFRB) [53], which have not been tested in this study. Additionally, we are able to provide detailed insight into the expression of a series of commonly used surface antigen markers of BS cells compared to BMSCs (Table 1) and give insights into the comparative transcriptome characteristics between these two cell types (Fig. 2). Interestingly, BS cells have also been shown to be able to undergo neurogenic [54] and tendogenic differentiation [55] underlining their potential impact for the treatment of subacromial pathology. Because of the similarities between both cell types, as shown in this work, it is also conceivable that BMSCs, which can be easily obtained from bone marrow, might be harnessed to augment reconstitution of the subacromial bursa after surgical removal, providing potential support for the restoration of the gliding mechanism after such treatments.

## Conclusions

The present study shows that cells isolated from the subacromial bursa of the shoulder meet the minimal criteria for their classification as MSCs [56]. Although in certain areas, marked differences between BS cells and BMSCs could be resolved on molecular levels (e.g. ALP expression, transcriptome), both cell types could be expanded using plastic adherence, are capable of multilineage differentiation, and showed a similar expression of several MSC surface markers, indicating the MSC potential of the BS cells. Such multipotent BS cells can be located at high density in the fibrous, as well as the mucinous parts of the BS tissue. Thus, we conclude that BS tissues can be regarded as a reservoir rich in MSCs within the shoulder, and it remains to be seen in further investigations, whether this knowledge might be harnessed for the development of improved treatments for subacromial pathologies.

## Abbreviations

A: Antisense
AGN: Aggrecan
ALP: Alkaline phosphatase
APC: Allophycocyanin
BSP: Integrin-binding sialoprotein
BMSC: Bone marrow-derived MSC
Cbfa1: Core binding factor alpha 1
CD: Cluster of differentiation
COL I: Collagen type I
COL II: Collagen type II
DEC: Decorin
EF1α: Elongation factor 1α
FGF: Fibroblast growth factor
FITC: Fluorescein isothiocyanate
FM: Fibromodulin
FOXP2: Forkhead box P2
IHH: Indian hedgehog
LPL: Lipoprotein lipase
Meox: Mesenchyme homeobox 2
MSC: Mesenchymal stem cell
MUC1: Mucin1
PE: Phycoerythrin
PPARγ2: Peroxisome proliferator-activated receptor gamma 2
PRG4: Proteoglycan 4
S: Sense
SOX9: SRY (sex determining region Y)-box 9
temp.: Temperature
WISP3: WNT1 inducible signalling pathway protein 3

## References

[1] Gohlke F, Rolf O, Bohm D (2007). Open reconstruction of the rotator cuff. Orthopade.

[2] Gohlke F (1993). Ultrasonographic appearance of the rotator cuff in elderly subjects. Orthopade.

[3] Cofield RH, Parvizi J, Hoffmeyer PJ, Lanzer WL, Ilstrup DM, Rowland CM (2001). Surgical repair of chronic rotator cuff tears. A prospective long-term study. J Bone Joint Surg Am.

[4] Harryman DT, Hettrich CM, Smith KL, Campbell B, Sidles JA, Matsen FA (2003). A prospective multipractice investigation of patients with full-thickness rotator cuff tears: the importance of comorbidities, practice, and other covariables on self-assessed shoulder function and health status. J Bone Joint Surg Am.

[5] Rolf O, Ochs K, Bohm TD, Baumann B, Kirschner S, Gohlke F (2006). Rotator cuff tear–an occupational disease? An epidemiological analysis. Z Orthop Ihre Grenzgeb.

[6] Loew M, Habermeyer P, Wiedemann E, Rickert M, Gohlke F (2000). Recommendations for diagnosis and expert assessment of traumatic rotator cuff lesions. Unfallchirurg.

[7] Jacobs J (2008). The burden of musculoskeletal diseases in the United States.

[8] Ishii H, Brunet JA, Welsh RP, Uhthoff HK (1997). “Bursal reactions” in rotator cuff tearing, the impingement syndrome, and calcifying tendinitis. J Shoulder Elbow Surg.

[9] Sarkar K, Uhthoff HK (1983). Ultrastructure of the subacromial bursa in painful shoulder syndromes. Virchows Arch A Pathol Anat Histopathol.

[10] Birnbaum K, Lierse W (1992). Anatomy and function of the bursa subacromialis. Acta Anat (Basel).

[11] Roepcke S, Stahlberg S, Klein H, Schulz MH, Theobald L, Gohlke S (2011). A tandem sequence motif acts as a distance-dependent enhancer in a set of genes involved in translation by binding the proteins NonO and SFPQ. BMC Genomics.

[12] Gotoh M, Hamada K, Yamakawa H, Inoue A, Fukuda H (1998). Increased substance P in subacromial bursa and shoulder pain in rotator cuff diseases. J Orthop Res.

[13] Blaine TA, Kim YS, Voloshin I, Chen D, Murakami K, Chang SS (2005). The molecular pathophysiology of subacromial bursitis in rotator cuff disease. J Shoulder Elbow Surg.

[14] Voloshin I, Gelinas J, Maloney MD, O’Keefe RJ, Bigliani LU, Blaine TA. Proinflammatory cytokines and metalloproteases are expressed in the subacromial bursa in patients with rotator cuff disease. Arthroscopy. 2005;21:1076 e1071–9. 10.1016/j.arthro.2005.05.017 16171632

[15] Uhthoff HK, Sarkar K (1991). Surgical repair of rotator cuff ruptures. The importance of the subacromial bursa. J Bone Joint Surg Br.

[16] Uhthoff HK, Sano H, Trudel G, Ishii H (2000). Early reactions after reimplantation of the tendon of supraspinatus into bone. A study in rabbits. J Bone Joint Surg Br.

[17] Hirose K, Kondo S, Choi HR, Mishima S, Iwata H, Ishiguro N (2004). Spontaneous healing process of a supraspinatus tendon tear in rabbits. Arch Orthop Trauma Surg.

[18] Iwata Y, Morihara T, Tachiiri H, Kajikawa Y, Yoshida A, Arai Y (2008). Behavior of host and graft cells in the early remodeling process of rotator cuff defects in a transgenic animal model. J Shoulder Elbow Surg.

[19] Neuwirth J, Fuhrmann RA, Veit A, Aurich M, Stonans I, Trommer T (2006). Expression of bioactive bone morphogenetic proteins in the subacromial bursa of patients with chronic degeneration of the rotator cuff. Arthritis Res Ther.

[20] Friedenstein AJ, Chailakhjan RK, Lalykina KS (1970). The development of fibroblast colonies in monolayer cultures of guinea-pig bone marrow and spleen cells. Cell Tissue Kinet.

[21] Pittenger MF, Mackay AM, Beck SC, Jaiswal RK, Douglas R, Mosca JD (1999). Multilineage potential of adult human mesenchymal stem cells. Science.

[22] Noth U, Osyczka AM, Tuli R, Hickok NJ, Danielson KG, Tuan RS (2002). Multilineage mesenchymal differentiation potential of human trabecular bone-derived cells. J Orthop Res.

[23] Zuk PA, Zhu M, Mizuno H, Huang J, Futrell JW, Katz AJ (2001). Multilineage cells from human adipose tissue: implications for cell-based therapies. Tissue Eng.

[24] Tallheden T, Dennis JE, Lennon DP, Sjogren-Jansson E, Caplan AI, Lindahl A (2003). Phenotypic plasticity of human articular chondrocytes. J Bone Joint Surg Am.

[25] Jankowski RJ, Deasy BM, Huard J (2002). Muscle-derived stem cells. Gene Ther.

[26] Bi Y, Ehirchiou D, Kilts TM, Inkson CA, Embree MC, Sonoyama W (2007). Identification of tendon stem/progenitor cells and the role of the extracellular matrix in their niche. Nat Med.

[27] Salingcarnboriboon R, Yoshitake H, Tsuji K, Obinata M, Amagasa T, Nifuji A (2003). Establishment of tendon-derived cell lines exhibiting pluripotent mesenchymal stem cell-like property. Exp Cell Res.

[28] Steinert AF, Kunz M, Prager P, Barthel T, Jakob F, Noth U (2011). Mesenchymal stem cell characteristics of human anterior cruciate ligament outgrowth cells. Tissue Eng Part A.

[29] Cheng MT, Yang HW, Chen TH, Lee OK (2009). Isolation and characterization of multipotent stem cells from human cruciate ligaments. Cell Prolif.

[30] Huang TF, Chen YT, Yang TH, Chen LL, Chiou SH, Tsai TH (2008). Isolation and characterization of mesenchymal stromal cells from human anterior cruciate ligament. Cytotherapy.

[31] De Bari C, Dell’Accio F, Vanlauwe J, Eyckmans J, Khan IM, Archer CW (2006). Mesenchymal multipotency of adult human periosteal cells demonstrated by single-cell lineage analysis. Arthritis Rheum.

[32] Mauck RL, Martinez-Diaz GJ, Yuan X, Tuan RS (2007). Regional multilineage differentiation potential of meniscal fibrochondrocytes: implications for meniscus repair. Anat Rec (Hoboken).

[33] Solchaga LA, Penick K, Porter JD, Goldberg VM, Caplan AI, Welter JF (2005). FGF-2 enhances the mitotic and chondrogenic potentials of human adult bone marrow-derived mesenchymal stem cells. J Cell Physiol.

[34] Limbert C, Ebert R, Schilling T, Path G, Benisch P, Klein-Hitpass L (2010). Functional signature of human islet-derived precursor cells compared to bone marrow-derived mesenchymal stem cells. Stem Cells Dev.

[35] Tusher VG, Tibshirani R, Chu G (2001). Significance analysis of microarrays applied to the ionizing radiation response. Proc Natl Acad Sci U S A.

[36] Rainer J, Sanchez-Cabo F, Stocker G, Sturn A, Trajanoski Z (2006). CARMAweb: comprehensive R- and bioconductor-based web service for microarray data analysis. Nucleic Acids Res.

[37] Johnstone B, Hering TM, Caplan AI, Goldberg VM, Yoo JU (1998). In vitro chondrogenesis of bone marrow-derived mesenchymal progenitor cells. Exp Cell Res.

[38] Sakaguchi Y, Sekiya I, Yagishita K, Muneta T (2005). Comparison of human stem cells derived from various mesenchymal tissues: superiority of synovium as a cell source. Arthritis Rheum.

[39] Djouad F, Jackson WM, Bobick BE, Janjanin S, Song Y, Huang GT (2010). Activin A expression regulates multipotency of mesenchymal progenitor cells. Stem Cell Res Ther.

[40] Segawa Y, Muneta T, Makino H, Nimura A, Mochizuki T, Ju YJ (2009). Mesenchymal stem cells derived from synovium, meniscus, anterior cruciate ligament, and articular chondrocytes share similar gene expression profiles. J Orthop Res.

[41] Iwata T, Yamato M, Zhang Z, Mukobata S, Washio K, Ando T (2010). Validation of human periodontal ligament-derived cells as a reliable source for cytotherapeutic use. J Clin Periodontol.

[42] Lin G, Garcia M, Ning H, Banie L, Guo YL, Lue TF (2008). Defining stem and progenitor cells within adipose tissue. Stem Cells Dev.

[43] Gronthos S, Franklin DM, Leddy HA, Robey PG, Storms RW, Gimble JM (2001). Surface protein characterization of human adipose tissue-derived stromal cells. J Cell Physiol.

[44] Kopher RA, Penchev VR, Islam MS, Hill KL, Khosla S, Kaufman DS (2010). Human embryonic stem cell-derived CD34+ cells function as MSC progenitor cells. Bone.

[45] Lippross S, Loibl M, Hoppe S, Meury T, Benneker L, Alini M (2011). Platelet released growth factors boost expansion of bone marrow derived CD34(+) and CD133(+) endothelial progenitor cells for autologous grafting. Platelets.

[46] Untergasser G, Koeck R, Wolf D, Rumpold H, Ott H, Debbage P (2006). CD34+/CD133- circulating endothelial precursor cells (CEP): characterization, senescence and in vivo application. Exp Gerontol.

[47] Schilling T, Kuffner R, Klein-Hitpass L, Zimmer R, Jakob F, Schutze N (2008). Microarray analyses of transdifferentiated mesenchymal stem cells. J Cell Biochem.

[48] Fakhry A, Ratisoontorn C, Vedhachalam C, Salhab I, Koyama E, Leboy P (2005). Effects of FGF-2/-9 in calvarial bone cell cultures: differentiation stage-dependent mitogenic effect, inverse regulation of BMP-2 and noggin, and enhancement of osteogenic potential. Bone.

[49] Yamaoka H, Nishizawa S, Asawa Y, Fujihara Y, Ogasawara T, Yamaoka K (2010). Involvement of fibroblast growth factor 18 in dedifferentiation of cultured human chondrocytes. Cell Prolif.

[50] Koning N, van Eijk M, Pouwels W, Brouwer MS, Voehringer D, Huitinga I (2010). Expression of the inhibitory CD200 receptor is associated with alternative macrophage activation. J Innate Immun.

[51] Blaine TA, Cote MA, Proto A, Mulcahey M, Lee FY, Bigliani LU (2011). Interleukin-1beta stimulates stromal-derived factor-1alpha expression in human subacromial bursa. J Orthop Res.

[52] Ko JY, Wang FS, Huang HY, Wang CJ, Tseng SL, Hsu C (2008). Increased IL-1beta expression and myofibroblast recruitment in subacromial bursa is associated with rotator cuff lesions with shoulder stiffness. J Orthop Res.

[53] Lhee SH, Jo YH, Kim BY, Nam BM, Nemeno JG, Lee S (2013). Novel supplier of mesenchymal stem cell: subacromial bursa. Transplant Proc.

[54] Aydin A, Duruksu G, Erman G, Subasi C, Aksoy A, Unal ZS (2014). Neurogenic differentiation capacity of subacromial bursal tissue-derived stem cells. J Orthop Res.

[55] Song N, Armstrong AD, Li F, Ouyang H, Niyibizi C (2014). Multipotent mesenchymal stem cells from human subacromial bursa: potential for cell based tendon tissue engineering. Tissue Eng Part A.

[56] Dominici M, Le Blanc K, Mueller I, Slaper-Cortenbach I, Marini F, Krause D (2006). Minimal criteria for defining multipotent mesenchymal stromal cells. The International Society for Cellular Therapy position statement. Cytotherapy.

